# Developmental changes in mental rotation ability and visual perspective-taking in children and adults with Williams syndrome

**DOI:** 10.3389/fnhum.2013.00856

**Published:** 2013-12-11

**Authors:** Masahiro Hirai, Yukako Muramatsu, Seiji Mizuno, Naoko Kurahashi, Hirokazu Kurahashi, Miho Nakamura

**Affiliations:** ^1^Department of Functioning Science, Institute for Developmental Research, Aichi Human Service CenterAichi, Japan; ^2^Department of Pediatrics, Central Hospital, Aichi Human Service CenterAichi, Japan

**Keywords:** Williams syndrome, visual perspective taking, mental rotation, developmental trajectory, children, developmental disorder, reference frame

## Abstract

Williams syndrome (WS) is a genetic disorder caused by the partial deletion of chromosome 7. Individuals with WS have atypical cognitive abilities, such as hypersociability and compromised visuospatial cognition, although the mechanisms underlying these deficits, as well as the relationship between them, remain unclear. Here, we assessed performance in mental rotation (MR) and level 2 visual perspective taking (VPT2) tasks in individuals with and without WS. Individuals with WS obtained lower scores in the VPT2 task than in the MR task. These individuals also performed poorly on both the MR and VPT2 tasks compared with members of a control group. For the individuals in the control group, performance scores improved during development for both tasks, while the scores of those in the WS group improved only in the MR task, and not the VPT2 task. Therefore, we conducted a second experiment to explore the specific cognitive challenges faced by people with WS in the VPT2 task. In addition to asking participants to change their physical location (self-motion), we also asked them to adopt a third-person perspective by imagining that they had moved to a specified location (self-motion imagery). This enabled us to assess their ability to simulate the movement of their own bodies. The performance in the control group improved in both the self-motion and self-motion imagery tasks and both performances were correlated with verbal mental age. However, we did not find any developmental changes in performance for either task in the WS group. Performance scores for the self-motion imagery task in the WS group were low, similar to the scores observed for the VPT2 in this population. These results suggest that MR and VPT2 tasks involve different processes, and that these processes develop differently in people with WS. Moreover, difficulty completing VPT2 tasks may be partly because of an inability of people with WS to accurately simulate mental body motion.

## Introduction

Williams syndrome (WS) is a rare genetic disorder caused by the deletion of approximately 25 genes on chromosome 7. The prevalence of WS is between 1:20000 and 1:7500 (Stromme et al., 2002; Meyer-Lindenberg et al., 2006). Although there is heterogeneity in the cognitive domains that are affected by WS (Porter and Coltheart, 2005), several specific cognitive strengths and weaknesses have been consistently reported in this population (Bellugi et al., 2000; Meyer-Lindenberg et al., 2006; Martens et al., 2008; Riby and Porter, 2010; Jarvinen et al., 2013). For instance, the literature suggests that while language and auditory abilities are generally preserved (Bellugi et al., 1990; Karmiloff-Smith et al., 1997; Jordan et al., 2002; Brock, 2007), elements of visuospatial cognition, such as perceptual grouping, mental imagery, and global motion processing, are impaired (Bellugi et al., 1988; Pezzini et al., 1999; Farran et al., 2001; Nakamura et al., 2001; Atkinson et al., 2003, 2006; Hoffman et al., 2003; Farran and Jarrold, 2004, 2005). The observed deficits in visuospatial processing in people with WS may be due to atypical processing in the construction, but not the modality, of perception (Farran and Jarrold, 2003; Hoffman et al., 2003). Some evidence has also suggested that such visuospatial deficits extend to the memory domain (Vicari et al., 2003, 2005), and may, for instance, include the abnormal representation of reference frames (Nardini et al., 2008).

Neuroimaging research has indicated that visuospatial deficits in individuals with WS may be caused by a dysfunctional dorsal stream (Atkinson et al., 1997). Several atypical cortical structures have been observed in this population, such as (1) a low density of gray matter in the superior parietal regions (Reiss et al., 2004; Eckert et al., 2005), including the intraparietal sulcus (Meyer-Lindenberg et al., 2004), (2) bilateral reductions in the depth of the intraparietal/occipitotemporal sulci (Kippenhan et al., 2005) compared with controls, and (3) prominent folding abnormalities in the dorsal parietal cortex (Van Essen et al., 2006). Atypical fractional anisotropy in the right superior longitudinal fasciculus, which is associated with deficits in visuospatial construction, has also been reported in individuals with WS (Hoeft et al., 2007).

One prominent social phenotype of people with WS is that they display an empathetic nature and an extreme interest in both familiar and unfamiliar people. This particular trait has been termed “hypersociability” (Jones et al., 2000). Individuals with WS are often able to retrieve explicit emotional information from facial expressions (Gagliardi et al., 2003; Plesa-Skwerer et al., 2006; Skwerer et al., 2006) and perceive human actions from point-light motion (Jordan et al., 2002; Reiss et al., 2005; Hirai et al., 2009). However, the ability of this population to interpret emotional states seems to be atypical, such that they may have difficulty understanding unfamiliar facial expressions (Frigerio et al., 2006; Porter et al., 2007) or retrieving information about intent from motion (Van Der Fluit et al., 2012).

People with WS may have difficulty inferring the thoughts or emotions of others, although research on this issue has produced unclear results. An early WS study reported that individuals with this disorder perform well in a location change task (Karmiloff-Smith et al., 1995). Another study found that half of a group of people with WS performed similarly to normal adults on a task where participants were asked to identify complex emotional states from photographs of eyes (Tager-Flusberg et al., 1998). However, later studies of children with WS found impaired mentalizing ability (Tager-Flusberg et al., 1997; Sullivan and Tager-Flusberg, 1999; Tager-Flusberg and Sullivan, 2000). Porter et al. (2008) reported a specific deficit in social understanding in one of two WS subgroups, indicated by poor performance on a non-verbal version of the theory of mind (ToM) task. This effect persisted even when the effects of mental or chronological age were removed. This finding suggests cognitive heterogeneity in the social cognition of individuals with WS (Porter and Coltheart, 2005).

Accumulating evidence shows that individuals with WS have atypical cognitive abilities, such as hypersociability and impaired visuospatial cognition. However, the mechanisms underlying these deficits are unclear, as is the relationship between impaired social cognition and impaired visuospatial cognition.

Visual perspective taking tasks can be used to assess connections between visuospatial and social cognitive processes. Visual perspective taking has two levels: Level 1 visual perspective taking (VPT1) refers to knowledge about which objects in one's frame of view are visible to another observer, while Level 2 visual perspective taking (VPT2) refers to the knowledge that two different observers can have unique visual experiences of the same scene or object (Flavell et al., 1984). Developmental psychological studies have shown that both levels are not acquired simultaneously. Infants are first able to understand VPT1 at approximately 24 months (Moll and Tomasello, 2006). It is not until later, in the preschool period, that individuals are able to understand VPT2 (Flavell, 1999). For instance, a recent study reported that 3-year-old children are able to successfully complete a VPT2 task (Moll and Meltzoff, 2011).

Several studies have investigated the connection between different characteristics of cognitive tasks. For instance, one behavioral study reported a clear relationship between the performance of children aged 4–8 years on a ToM and a VPT2 task, but not between a ToM and a mental rotation (MR) task (Hamilton et al., 2009). This suggests that ToM and VPT2 tasks may have common cognitive processes that may not be required for MR tasks. Therefore, the VPT2 task may be useful in assessing mentalizing ability in individuals with WS. The notion that ToM and VPT2 tasks may have common cognitive processes has been supported by several neuroimaging findings. For instance, in adults, the temporoparietal junction (TPJ) is activated by VPT2 tasks (Zacks et al., 2003b; Aichhorn et al., 2006) and false-belief tasks (Saxe and Kanwisher, 2003). The importance of the TPJ for performance on the above-mentioned tasks has been demonstrated by lesion studies (Apperly et al., 2004) and transcranial direct current stimulation studies (Santiesteban et al., 2012). However, these studies reported no overlap in terms of the neural activities underlying the VPT2 and MR tasks, indicating that differential brain networks are involved.

The current study comprised two experiments. The first focused on developmental changes in MR and VPT2 task performance in individuals with WS, and employed tasks developed by Hamilton et al. (2009). In Experiment 1, we hypothesized that, (1) in light of previous findings regarding deficient visuospatial skills in individuals with WS, this population would have impaired MR ability compared with normal controls, and (2) if individuals with WS exhibited impaired mentalizing ability (Tager-Flusberg et al., 1997; Tager-Flusberg and Sullivan, 2000; Porter and Coltheart, 2005; Porter et al., 2008), then VPT2 task performance would be poor compared with normal controls.

In our preliminary experiment, we found that members of the WS group consistently had difficulties completing the VPT2 task. Therefore, our second experiment was designed to explore the nature of these difficulties. Although a recent neuroimaging study has demonstrated that different brain regions are involved in the spatial transformation of oneself vs. another person (Mazzarella et al., 2013), behavioral evidence suggests that spatial perspective taking is an embodied cognitive process, in the sense that the participant's own body posture can interfere with performance on a VPT2 task. This implies that cognitive processes underlying spatial transformation of oneself and of others may overlap (e.g., Kessler and Thomson, 2010). Thus, differential performance on VPT2 and spatial transformation tasks could help to explain the difficulty observed in the VPT2 task in Experiment 1.

In Experiment 2, we manipulated the location of the participants with respect to an object (first-person location). We asked the participants to either move to a new position or to imagine that they had moved. Both manipulations were designed to match the difficulty of the procedure in Experiment 1. If the expected difficulties in VPT2 task completion in Experiment 1 were due to defective mental body motion simulation in people with WS, then this would reflect performance on the self-motion imagery task.

## Materials and methods (Experiment 1)

### Participants

Twenty-six people with WS (13 males and 13 females) participated in the experiments (Table 1). Twenty participants were recruited from our institute, and six were recruited through the Williams Syndrome Association in Aichi prefecture (Elfin Chubu, Nagoya). All participants had been phenotypically diagnosed by clinicians, with their diagnoses confirmed through positive fluorescence *in situ* hybridization testing. The ages of the participants ranged from 6 years 0 months to 33 years 5 months (mean age = 16 years and 2 months). Verbal intelligence was measured with the Japanese version of the Picture Vocabulary Scale (JPVS) (Ueno et al., 2008).

**Table 1:** **Participants**.

**Group**	***N* (F/M)**	**Chronological age mean (year) range (years; months)**	**Verbal mental age mean (year) range (years; months)**
WS	26 (13/13)	16.2 ± 7.2 (6;0–33;4)	7.46 ± 2.46 (3;3–11;1)
VMA	26 (13/13)	7.67 ± 2.7 (3;9–13;2)	7.62 ± 2.5 (3;5–12;3)
CA	26 (13/13)	16.3 ± 8.4 (6;5–40;6)	N/A

(Mean ± SD).

Fifty-two typically developed children, adolescents, and adults were recruited from elementary schools, junior high schools, and universities near the institute as control groups (Table 1). For the verbal mental age-matched (VMA) group, 26 children (13 males) were selected to match individual JPVS scores obtained from participants with WS. For the chronological age-matched (CA) group, the ages of the control participants were individually matched to the ages of the participants with WS.

### Ethical considerations

All children, their parents, and adult participants provided informed consent. The study protocol was approved by the Ethics Committee at the Institute for Developmental Research in the Aichi Human Service Center.

### Theory of mind testing

As in previous studies (Tager-Flusberg and Sullivan, 2000; Hamilton et al., 2009), we conducted the location change task (Wimmer and Perner, 1983; Baron-Cohen et al., 1985) and the unexpected contents task (Hogrefe et al., 1986) prior to conducting the MR and VPT2 tasks in the WS and VMA groups. Both tasks were scored such that one point was given when a participant successfully completed a ToM task; otherwise the score remained at 0. Because all of the participants in the CA group were above 6 years of age, they easily passed the ToM tasks. Thus, we did not include their performance on these tasks in the analysis.

### Mental rotation task and level 2 visual perspective taking task

As in a previous study (Hamilton et al., 2009), we conducted two experimental tasks (MR and VPT2) in same session, with a short (a few minutes) break between them. We performed three familiarization trials to familiarize the participant with the experimental settings prior to the first session. At the beginning of each familiarization trial, a small toy (a dog) was placed on a square turntable, which had distinctly colored sides. The participant was shown a piece of paper in a transparent folder (to prevent any damage to the paper) with four pictures of the toy, taken from four perspectives (front, back, left, and right). The participant was then asked: “Which dog are you looking at?” The participant was instructed to point to the picture that matched the perspective of the toy as it appeared on the turntable. After the participant pointed to one of the four pictures, the toy was covered with a transparent bucket, and the participant was asked: “When I lift the bucket, which dog will you see?” If the participant made errors during the trials, the experimenter corrected them. We initially found that the familiarization task was difficult for young children with WS, so we decided to use a transparent bucket.

Following the familiarization session, we conducted six trials for each task (MR and VPT2). The task order was counterbalanced across participants. For each task, we put a toy in either a front or back position for three trials, and then in a profile position for three trials. We used six different toys (one for each task; car, dump truck, loading shovel, reindeer, panda, and owl) to prevent the participant from remembering the position of each toy, and to draw their attention to the toy during the experiment. The response sheet contained four pictures of each toy, taken from four perspectives. These were placed in a random order to exclude any response bias effects.

**Figure 1:**
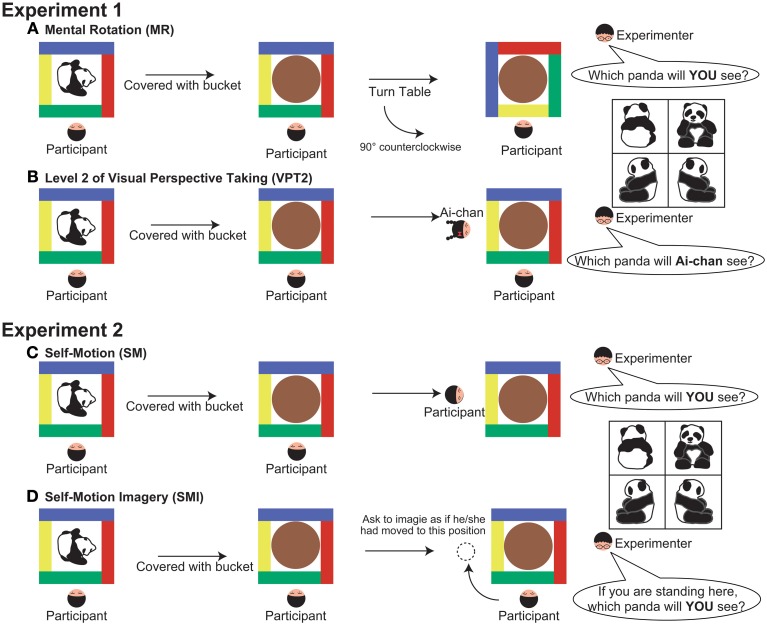
**Detail of the experimental procedure (a top-down view). (A)** mental rotation (MR), **(B)** level 2 of visual perspective taking (VPT2), **(C)** self-motion (SM), and **(D)** self-motion-imagery (SMI). In all experimental conditions, the experimenter began by confirming that the participants were attending to the orientation of the toy. After covering the bucket and completing the experimental manipulation, the experimenter asked the participants to point to one of the pictures on the response sheet, shown at the right side of the figure.

For the MR task, the experimenter told each participant to “watch carefully” and then placed a new toy on the table. The experimenter then showed the participant the response sheet and asked them to point to the picture that matched the position of the toy. This ensured that the participant was paying attention to the toy. The experimenter covered the toy with an opaque bucket and turned the table 90° clockwise, 180°, or 90° counter-clockwise. After turning the table, the experimenter asked the child: “If I lift the bucket, which “*toy name*” (i.e., “Panda” in Figure 1A) will you see?” The participant was instructed to point to the picture that they thought matched the position of the toy (Figure 1A).

For the VPT2 task, the experimenter placed a toy on the table and told the participant to “watch carefully.” The experimenter then gave the participant the response sheet and asked them to point to the picture that matched that position of the toy. The experimenter covered the toy with the opaque bucket, took out a doll from behind their back, and placed it on the left, right, or far side of the table, away from the participant. The experimenter then shook the doll side to side to draw the participant's attention, and asked: “This is Ai-chan; when I lift the bucket, which “*toy name*” (i.e., “Panda” in Figure 1B) will Ai-chan see?” Emphasis was put on the word “Ai-chan” when asking the question. The experimenter asked the participant to point to the picture that matched the perspective of the toy that the doll would see (Figure 1B).

The experiment was performed in a quiet playroom at our institute. During the sessions, the experimenter provided motivational feedback to the participant (e.g., “You are doing well!”) to keep their attention focused on the task, irrespective of their responses. We did not give any feedback regarding accuracy to the participants, and the experimenter told the participants that there was no time limit within which they had to respond.

### Data analysis

We counted the number of participants who successfully completed each ToM task. Chi-square analysis was used to assess performance across groups.

As per previous studies, we focused on correct answer responses (Hamilton et al., 2009) and error responses (e.g., Samson et al., 2007) when analyzing the data from Experiment 1. Our preliminary observations suggested that younger children tend to show an egocentric bias (i.e., even when the doll was placed in a different position, their response was identical to the response they gave before the toy was covered with the bucket) during the VPT2 task, as previously depicted in the three-mountain paradigm (Piaget and Inhelder, 1956). We defined this type of error as “egocentric-bias error”; and any other error was defined as a “non-egocentric-bias error.” In our analysis, we calculated the proportion of egocentric errors (the proportion of egocentric errors made in relation to the overall number of errors).

For statistical analysis, we applied a two-way mixed-design repeated measures analysis of variance (ANOVA) to the correct answers and the proportion of egocentric-bias errors. Group (WS, VMA, and CA groups) was used as a between-subject factor, and Task (MR and VPT2) was used as a within-subject factor.

We also analyzed correct responses based on performance in the two ToM tasks. In this analysis, we focused on the data from the WS and VMA groups, because the participants in the CA group were all older than 6 years, as mentioned above. We defined participants who passed both ToM tasks (i.e., the score was 2 points) as members of the ToM pass group. The two ToM tasks had similar levels of difficulty, and so a participant who passed one but not the other may just have been guessing. We applied a Three-Way mixed-design repeated measures ANOVA to the correct responses. Group (WS and VMA) and ToM performance (Pass group and Fail group) were used as between-subject factors, and Task (MR and VPT2) was used as a within-subject factor.

If the sphericity assumption was violated, as indicated by Mauchly's sphericity test, then the Greenhouse–Geisser epsilon coefficient was used to correct the degrees of freedom. Tukey's honestly significant difference test was applied for multiple comparisons. The *F* and *P*-values were then recalculated. A *P*-value of < 0.05 was considered statistically significant.

In addition to these analyses, we adopted a developmental trajectory approach (Thomas et al., 2009) to assess developmental changes in task performance in both the WS and VMA groups. We did not include the CA group in this analysis because their performance scores reached a ceiling level, and therefore, further developmental changes could not be observed. For this analysis, we calculated coefficients and evaluated improvements in performance based on developmental changes in verbal mental age.

## Results (Experiment 1)

### Theory of mind testing

A comparison of the location change task scores from the three groups revealed a significant difference in performance [χ^2^_(1)_ = 4.16, *p* < 0.05]. Further binomial testing revealed that significantly more than half of the participants in the VMA group passed the test (*p* < 0.01), while this was not the case in the WS group (*p* = 0.17). A comparison of the unexpected contents task scores also revealed a significant difference in performance [χ^2^_(1)_ = 11.5, *p* < 0.01]. Further binomial testing revealed that significantly more than half of the participants in the VMA group passed the test (*p* < 0.01), while this was not the case in the WS group (*p* = 1.0). The results indicate that significantly more participants in the VMA group passed the ToM tasks compared with the WS group. Conversely, significantly more participants in the WS group failed the ToM tasks compared with the VMA group (Table 2).

**Table 2:** **Performance on two theory of mind tasks**.

**Group**	**Unexpected contents task**	**Location change task**
WS	9/26	13/26
VMA	22/26	21/26

(Number of participants who passed the task/ Number of participants).

### Mental rotation task and level 2 visual perspective taking task

**Figure 2:**
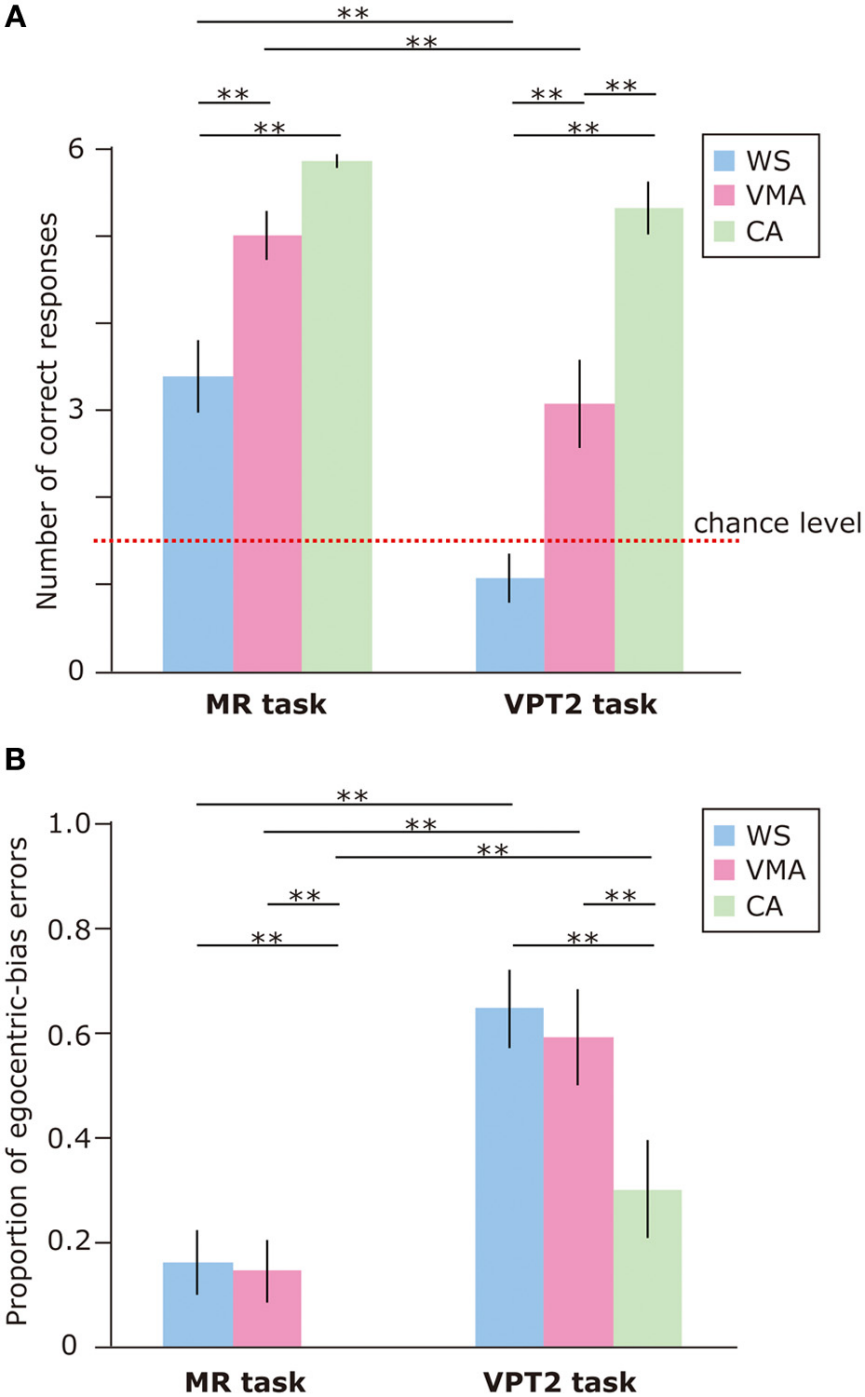
**(A)** Mean number of correct trials (max: 6) in the MR and VPT2 tasks for three groups [blue: Williams syndrome (WS) group; pink: verbal mental age-matched (VMA) group; green: chronological age-matched (CA) group]. **(B)** Mean proportion of egocentric errors (the proportion of egocentric errors made in relation to the overall errors) for both tasks. Error bars indicate standard error. ^**^p < 0.01.

To examine performance on the MR and VPT2 tasks, we first compared the number of correct responses in each group (Figure 2A). We observed significant effects of Group [*F*_(2, 75)_ = 39.8, *p* < 0.01] and Task [*F*_(1, 75)_ = 50.7, *p* < 0.01], and a significant two-way interaction between Group × Task [*F*_(2, 75)_ = 5.8, *p* < 0.01]. Subsequent follow-up analyses revealed that performance on the MR task was significantly greater than performance on the VPT2 task for participants in the WS [*F*_(1, 75)_ = 35.6, *p* < 0.01] and VMA [*F*_(1, 75)_ = 24.1, *p* < 0.01] groups. No significant differences were observed in the CA group [*F*_(1, 75)_ = 1.9, *p* = 0.17].

In terms of group differences, we observed that the performance of the WS group was worse than the performance of the VMA (*p* < 0.01) and CA groups (*p* < 0.01) on the MR task, although we found no difference between the VMA and CA groups (*p* = 0.07). For the VPT2 task, performance scores from the CA group were significantly better than scores from the VMA (*p* < 0.01) and WS groups (*p* < 0.01). Performance scores from the VMA group were significantly better than performance scores from the WS group (*p* < 0.01).

In all groups, MR task scores were significantly above chance [CA: *t*_(25)_ = 60.2, *p* < 0.01; VMA: *t*_(25)_ = 12.9, *p* < 0.01; WS: *t*_(25)_ = 4.7, *p* < 0.01]. In contrast, the scores from the WS group on the VPT2 task were not significantly better than chance [*t*_(25)_ = 1.6, *p* = 0.13]. The scores from the VMA group on the VPT2 task [*t*_(25)_ = 3.2, *p* < 0.01] and CA [*t*_(25)_ = 12.8, *p* < 0.01] were significantly above chance.

We also examined the proportion of egocentric-bias errors (Figure 2B). The effects of Group [*F*_(2, 75)_ = 7.06, *p* < 0.01] and Task [*F*_(1, 75)_ = 59.2, *p* < 0.01] were significant, but the two-way interaction between Group × Task [*F*_(2, 75)_ = 1.10, *p* = 0.34] was not. This suggests that the proportion of egocentric-bias errors in the VPT2 task was significantly higher than that in the MR task, for all groups. In terms of group differences, the proportion of egocentric-bias errors in both the WS and VMA groups (*p* < 0.01) was significantly higher than that in the CA group, for both tasks. However, no significant differences were observed between the WS and VMA group.

**Figure 3:**
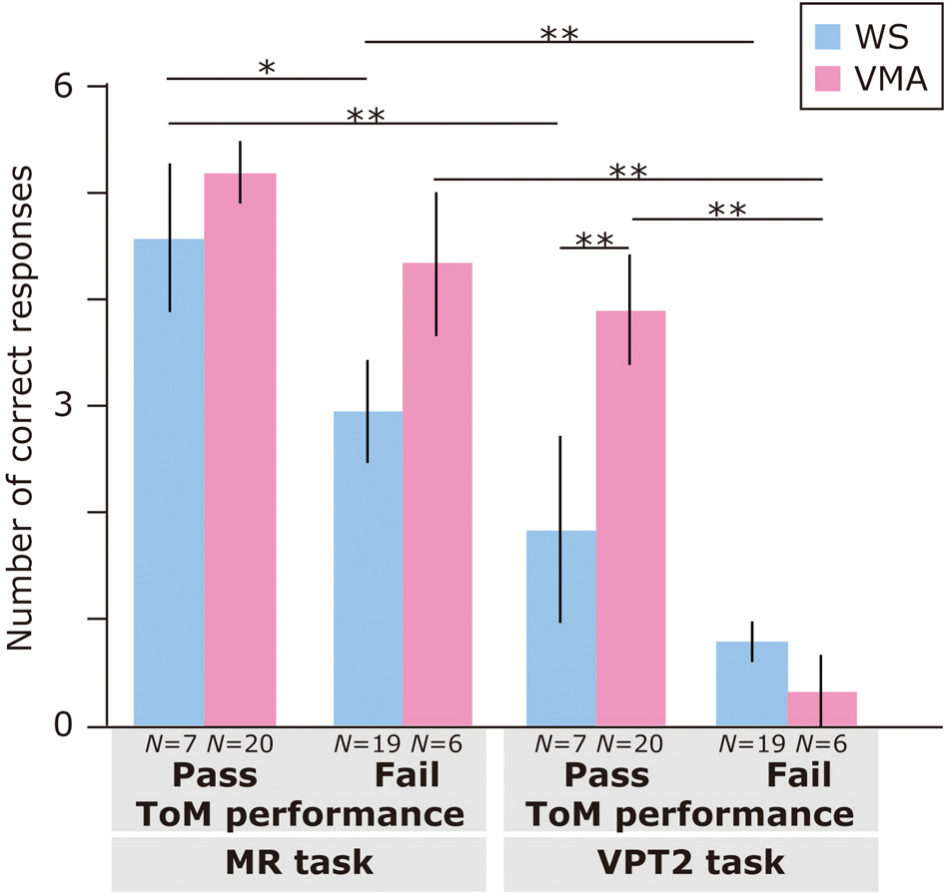
**Mean number of correct trials (max: 6) in the MR and VPT2 tasks based on ToM task performance of two groups [blue: Williams syndrome (WS) group; pink: verbal mental age-matched (VMA) group]**. ^**^p < 0.01; ^*^p < 0.05.

Regarding ToM task performance (Figure 3), we found that the main effects of Group [*F*_(1, 48)_ = 4.31, *p* < 0.05], ToM performance [*F*_(1, 48)_ = 16.9, *p* < 0.01], and Task [*F*_(1, 48)_ = 58.5, *p* < 0.01] were significant. Moreover, a three-way interaction of Group × ToM performance × Task [*F*_(1, 48)_ = 6.0, *p* < 0.01] was significant.

A follow-up analysis revealed that, for the VMA group, there were significantly more correct responses on the VPT2 task in the ToM pass group than in the ToM fail group [*F*_(1, 96)_ = 21.3, *p* < 0.01]. This was not the case for the MR task [*F*_(1, 96)_ = 1.26, *p* = 0.27]. For the WS group, there were significantly more correct responses on the MR task in the ToM pass group than in the ToM fail group [*F*_(1, 96)_ = 4.41, *p* < 0.05]. This effect was not observed for the VPT2 task [*F*_(1, 96)_ = 1.90, *p* = 0.17].

Regarding group differences, the VMA children who passed both ToM tasks had a significantly higher rate of correct VPT2 task performance than the individuals with WS who passed both ToM tasks [*F*_(1, 96)_ = 7.0, *p* < 0.01]. All other effects were not significant (all *Fs* < 3.2, *ps* > 0.08).

Regarding differences in performance across tasks, the WS group obtained significantly more correct answers in the MR task than in the VPT2 task, regardless of ToM task performance [ToM pass group: *F*_(1, 48)_ = 16.6, *p* < 0.01; ToM fail group: *F*_(1, 48)_ = 10.5, *p* < 0.01]. For the VMA participants, the above was true for the ToM fail group [*F*_(1, 48)_ = 36.2, *p* < 0.01], but not the ToM pass group [*F*_(1, 48)_ = 3.8, *p* = 0.06] in the VMA group.

**Figure 4:**
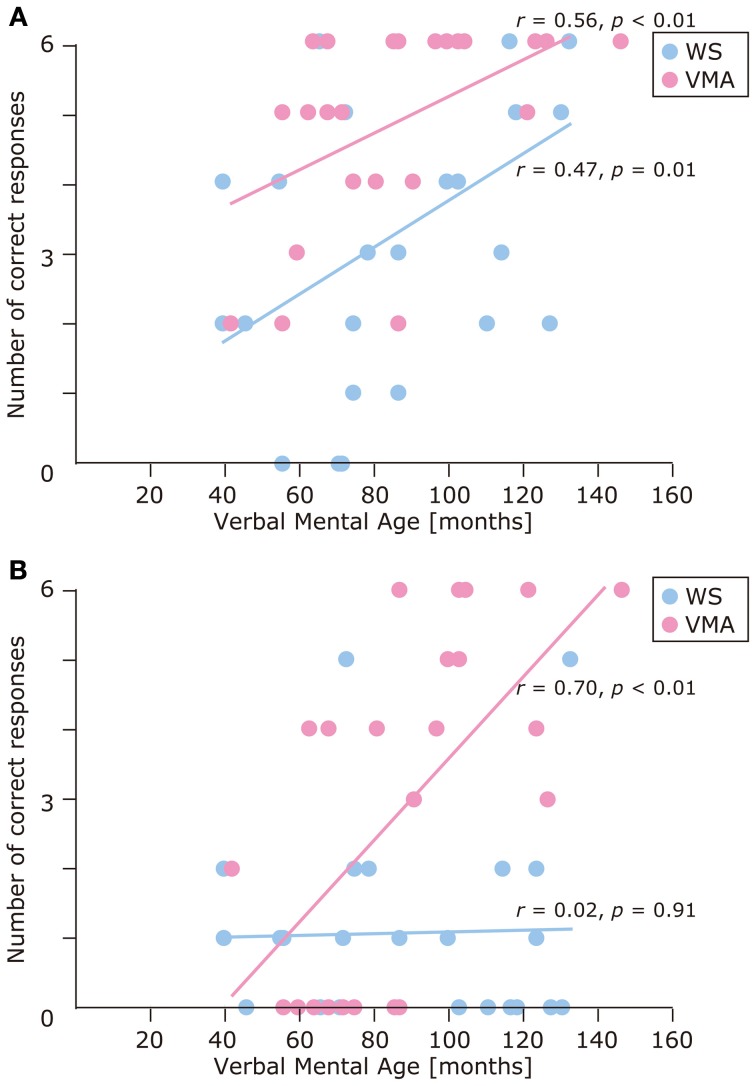
**Developmental trajectories for the number of correct responses in (A) the MR task, and (B) the VPT2 task, for two groups [blue: Williams syndrome (WS) group; pink: verbal mental age-matched (VMA) group]**.

**Figure 5:**
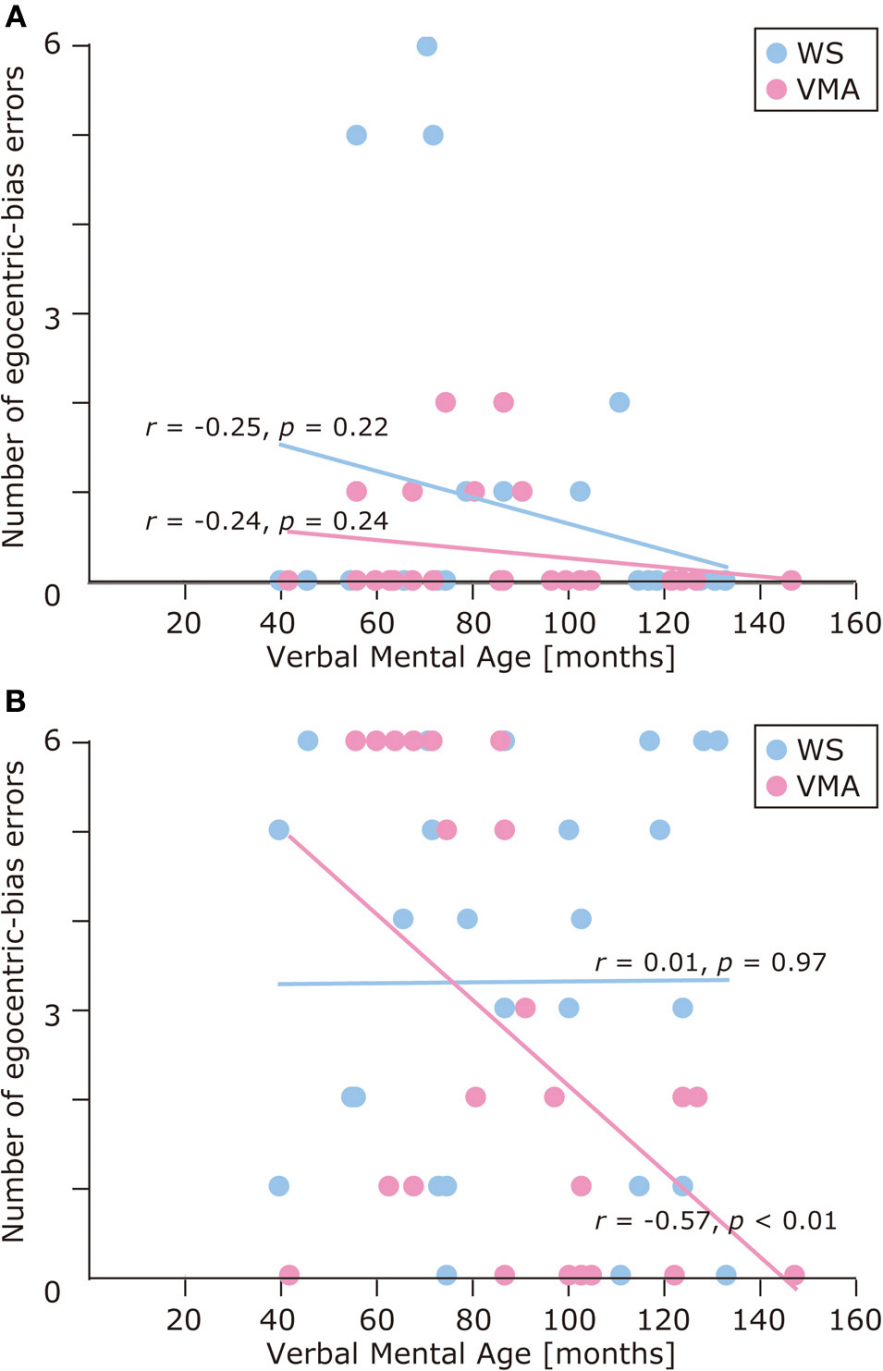
**Developmental trajectories for the number of egocentric-bias errors in (A) the MR task, and (B) the VPT2 task for two groups [blue: Williams syndrome (WS) group; pink: verbal mental age-matched (VMA) group]**.

We used a developmental trajectory approach to explore developmental changes in the WS and VMA groups in terms of correct and egocentric-bias error responses for both tasks (Figure 4). For the WS group, we observed a significant positive correlation between verbal mental age and performance on the MR (*r* = 0.47, *p* = 0.01) but not the VPT2 task (*r* = 0.02, *p* = 0.91). For the VMA group, we observed significant positive correlations between verbal mental age and performance for both the MR and VPT2 tasks (MR task; *r* = 0.56, *p* < 0.01; VPT2 task; *r* = 0.70, *p* < 0.01). In terms of egocentric-bias errors in the WS group, we did not observe any significant correlations (MR: *r* = −0.25, *p* = 0.22; VPT2: *r* = 0.01, *p* = 0.97). For the VMA group, we observed a significant negative correlation for the VPT2 (*r* = −0.57, *p* < 0.01) but not the MR task (*r* = −0.24, *p* = 0.24) (Figure 5).

## Materials and methods (Experiment 2)

### Participants

The participants that took part in Experiment 1 also took part in Experiment 2 (Table 1).

### Self-motion task and self-motion-imagery task

In Experiment 1, we found that the VPT2 task was more difficult for individuals with WS than the MR task. The performance of the WS group on the VPT2 task did not improve across development, in contrast with performance on the MR task. This motivated us to conduct a further experiment to explore alternative explanations for the observed difficulty, such as impaired mental simulation of one's own body motion. Behavioral evidence suggests that spatial perspective taking is an embodied cognitive process (Kessler and Thomson, 2010). Imagining one's own bodily motion can induce activation in distinct cortical regions, such as the left posterior parietal cortex (Creem et al., 2001), or supplementary motor areas (Wraga et al., 2005). Although these findings suggest that the demands of the VPT2 task include embodiment processes, it is likely that the neural activities involved in imagining one's own bodily motion are distinct from those activated by the VPT2. Thus, if we observed differential performance between VPT2 tasks and tasks requiring one to imagine the motion of their body, this might help to explain the difficulty observed in completing the VPT2 task in Experiment 1. To verify this possibility, we designed an experiment in which we manipulated the position (perspective) of the participant, instead of asking the participant to imagine a third-person perspective, as in Experiment 1. In Experiment 2, therefore, we introduced two experimental tasks, self-motion (SM) and self-motion imagery (SMI), in an attempt to match the task difficulty to that of Experiment 1.

For the SM condition, the experimenter placed a toy on a table and asked the participant to point to the picture on the response sheet (described in the methods for Experiment 1) that matched the position of the toy. This was done to make sure that the participant was paying attention to the toy. The experimenter then covered the toy with the opaque bucket and another experimenter gently took the participant's arms or shoulders to guide them in changing his or her location (to the left, right, or far side of the table with respect to the original position). After guiding the participant to the new position, the experimenter asked: “If I lift the bucket, which “*toy name*” (i.e., “Panda” in Figure 1C) will you see?” The participant was instructed to point to the picture that matched the perspective of the toy that they would see from their new position (Figure 1C).

For the SMI condition, the procedure was the same as in the SM condition, except that the experimenter pointed to a location (left, right, or far side of the table) instead of guiding the participant to that position. Before pointing to the location, the experimenter made sure that the participant understood the concept of imagining self-movement. The experimenter then asked the participant: “If you moved to this position and I lifted the bucket, which “*toy name*” (i.e., “Panda” in Figure 1D) would you see?” The participant was asked to point to the picture that matched the perspective of the toy that they would see from their new imagined position (Figure 1D).

Other than those detailed above, the experimental procedures were identical to those in Experiment 1. Six trials were performed for each task and the task order was counterbalanced across participants. The experiment was conducted in the same room as Experiment 1.

### Data analysis

As in Experiment 1, a Two-Way ANOVA was applied to the correct responses and the proportion of egocentric-bias errors. In the analysis, Group (WS, VMA, and CA groups) was used as a between-subject factor, and Task (SM and SMI) was used as a within-subject factor.

In addition to the ANOVA, we used the same methods as in Experiment 1 to analyze correct and incorrect ToM task responses for the WS and VMA groups. For each ToM task, a three-way mixed-design repeated measures ANOVA was applied to the correct responses. Group (WS and VMA) and ToM performance (Pass group and Fail group of participants) were used as between-subject factors, and Task (SM and SMI) was used as a within-subject factor. If the sphericity assumption was violated as per Mauchly's sphericity test, then the Greenhouse–Geisser epsilon coefficient was used to correct the degrees of freedom. Both the *F* and *P*-values were then recalculated. A *P*-value of < 0.05 was considered statistically significant.

In addition to these analyses, we adopted a developmental trajectory approach to assess developmental changes in task performance for both the WS and VMA groups (Thomas et al., 2009). As in Experiment 1, we did not apply this analysis to the CA group because their performance scores reached a ceiling level, thus, preventing further developmental changes from being observed. For this analysis, we calculated coefficients and evaluated improvements in performance based on developmental changes in verbal mental age.

## Results (Experiment 2)

**Figure 6:**
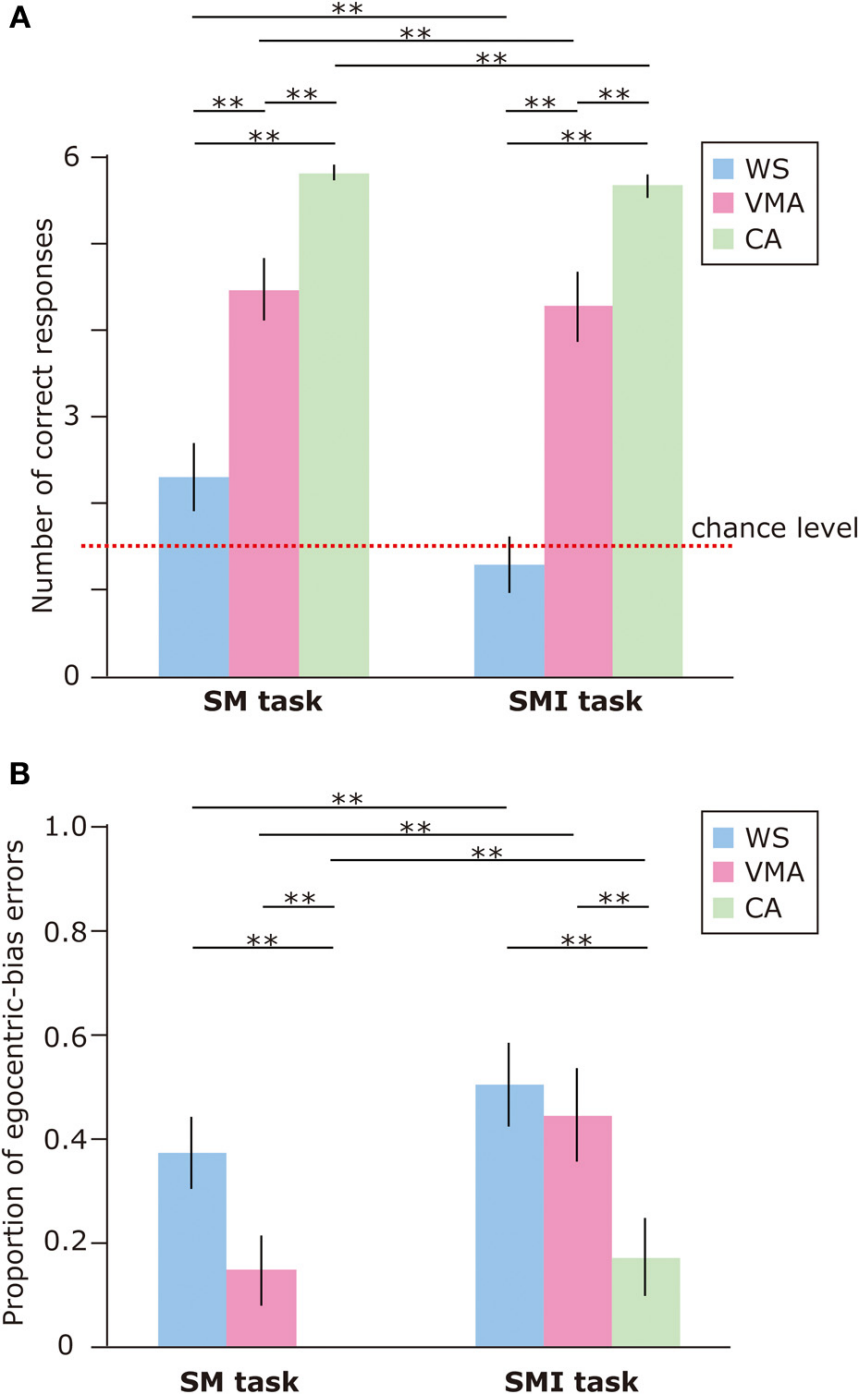
**(A)** Mean number of correct trials (max: 6) in the SM and SMI tasks for three groups [blue: Williams syndrome (WS) group; pink: verbal mental age-matched (VMA) group; green: chronological age-matched (CA) group]. **(B)** Mean proportion of egocentric errors (the proportion of egocentric errors made in relation to the overall errors) for both tasks. Error bars indicate standard error. ^**^p < 0.01.

To examine performance on the SM and SMI tasks, we applied an ANOVA to the number of correct responses (Figure 6A). We found that the effects of Group [*F*_(2, 75)_ = 59.8, *p* < 0.01] and Task [*F*_(1, 75)_ = 6.7, *p* < 0.05] were significant. A two-way interaction between Group × Task was marginally significant [*F*_(2, 75)_ = 2.5, *p* = 0.09]. This suggests that performance on the SM task was significantly better than performance on the SMI task, for all groups. With respect to group differences, the CA group performed significantly better than the VMA (*p* < 0.01) and WS (*p* < 0.01) groups. Performance in the VMA group was better than performance in the WS group (*p* < 0.01).

We also examined the proportion of egocentric-bias errors (Figure 6B). The effects of Group [*F*_(2, 75)_ = 10.4, *p* < 0.01] and Task [*F*_(1, 75)_ = 18.7, *p* < 0.01] were significant, but the two-way interaction between Group × Task [*F*_(2, 75)_ = 1.18, *p* = 0.31] was not. This indicates that the proportion of egocentric-bias errors was significantly higher in the SMI task than the SM task, for all groups. With respect to group differences, the proportion of egocentric-bias errors in both the WS (*p* < 0.01) and VMA (*p* < 0.01) groups was significantly higher than that in the CA group. However, no significant differences were observed between the WS and VMA groups.

In all groups, SM task performance was significantly above chance [CA: *t*_(25)_ = 54.7, *p* < 0.01; VMA: *t*_(25)_ = 8.52, *p* < 0.01; WS: *t*_(25)_ = 2.18, *p* < 0.05]. In contrast, the performance of the WS group on the SMI task was not significantly better than chance [*t*_(25)_ = 0.61, *p* = 0.54]. Performance in the VMA [*t*_(25)_ = 7.02, *p* < 0.01] and CA [*t*_(25)_ = 33.7, *p* < 0.01] groups on the SMI task was significantly better than chance.

**Figure 7:**
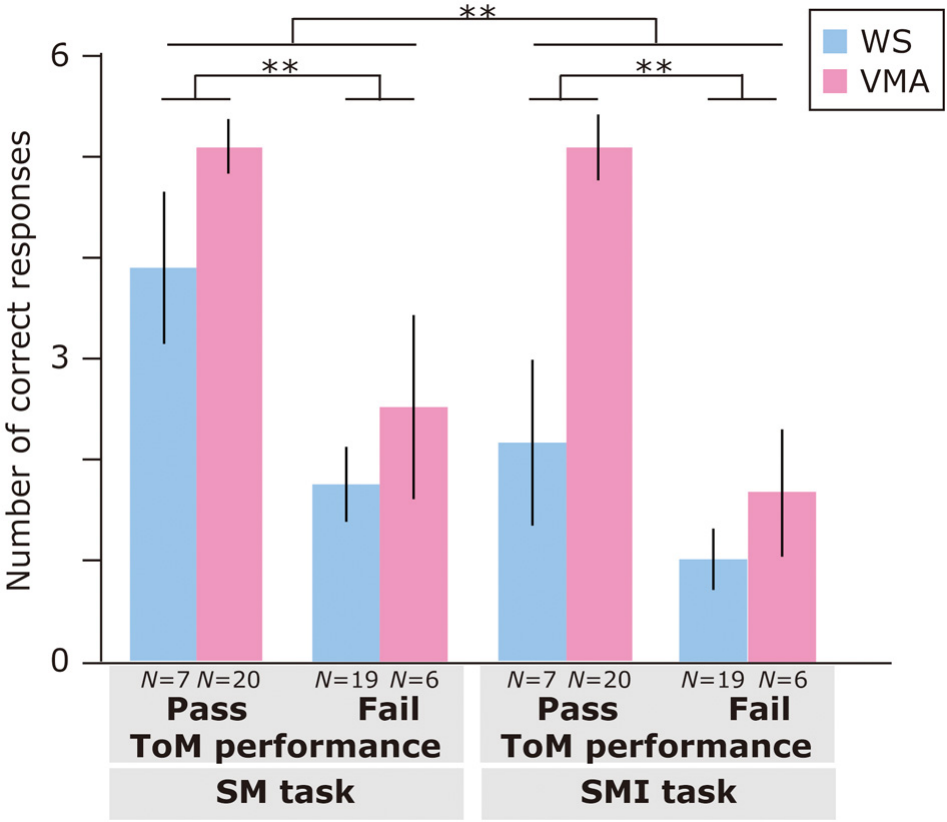
**Mean number of correct trials (max: 6) in the SM and SMI tasks based on ToM task performance for two groups [blue: Williams syndrome (WS) group; pink: verbal mental age-matched (VMA) group]**. Error bars indicate standard error. ^**^p < 0.01.

Regarding the relationship between ToM task performance and the number of correct responses (Figure 7), we found a significant main effects of Group [*F*_(1, 48)_ = 12.5, *p* < 0.01], ToM performance [*F*_(1, 48)_ = 34.6, *p* < 0.01], and Task [*F*_(1, 48)_ = 8.06, *p* < 0.01]. No other interactions were significant [all *Fs* < 2.9, *ps* > 0.09]. This suggests that the VMA group performed significantly better than the WS group, and that the members of the ToM pass group performed significantly better than the members of the ToM fail group. Moreover, SM task performance was significantly greater than SMI task performance.

**Figure 8:**
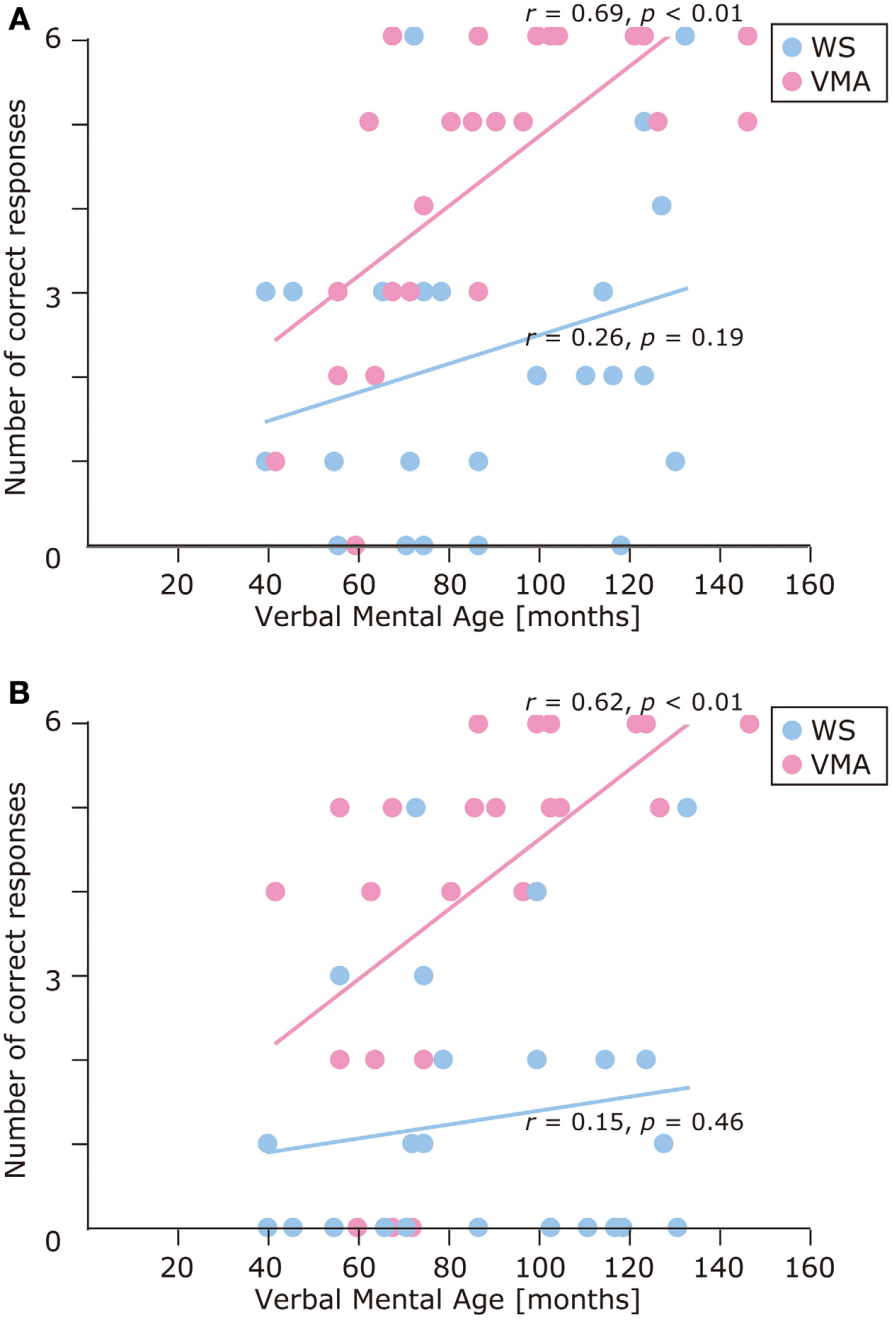
**Developmental trajectories for the number of correct responses in (A) the SM task, and (B) the SMI task for two groups [blue: Williams syndrome (WS) group; pink: verbal mental age-matched (VMA) group]**.

**Figure 9:**
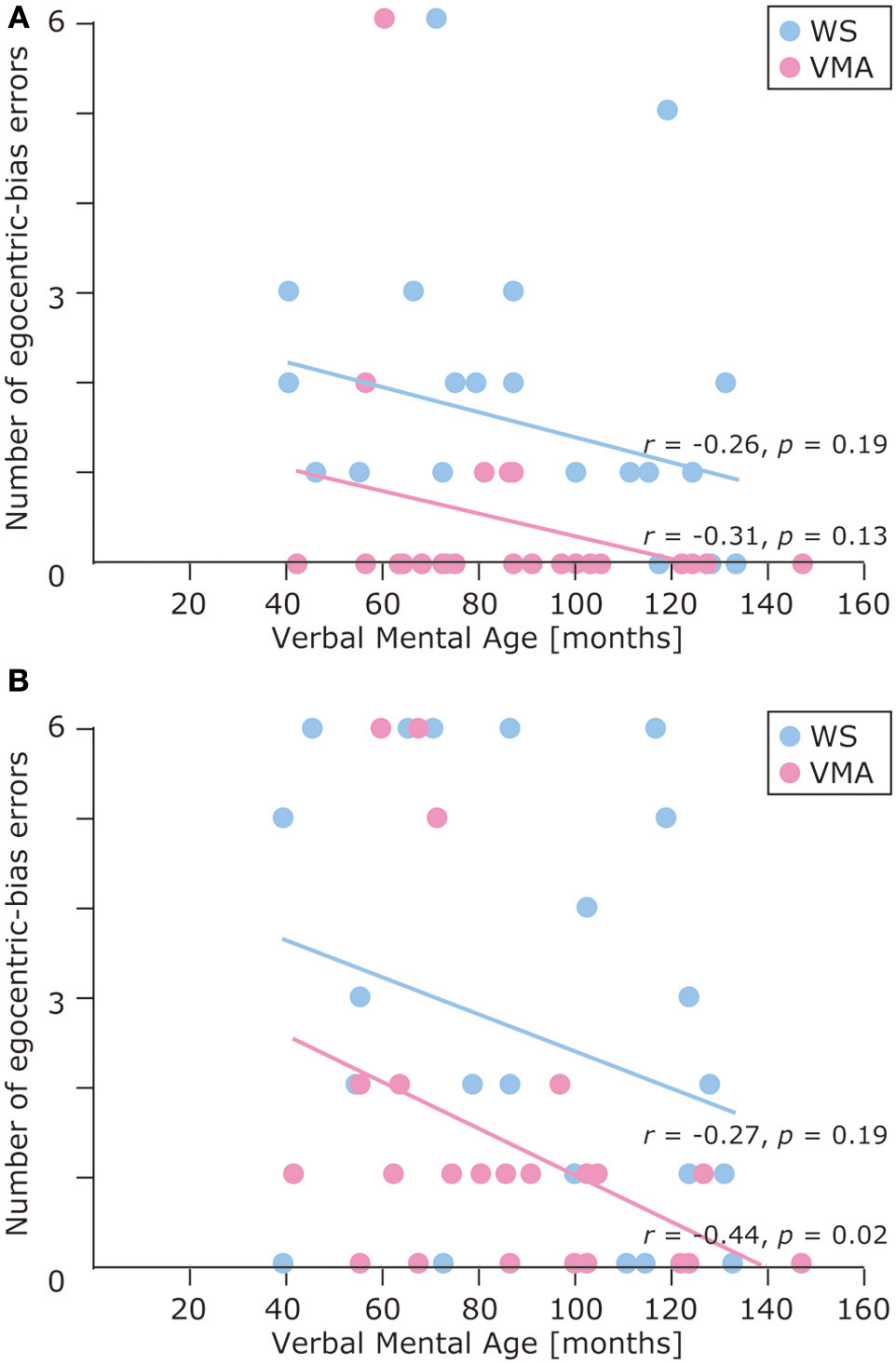
**Developmental trajectories for the number of egocentric-bias errors in (A) the SM task and (B) the SMI task for two groups [blue: Williams syndrome (WS) group; pink: verbal mental age-matched (VMA) group]**.

The results of the developmental trajectory analysis indicated significant positive correlations between verbal mental age and correct performance in the VMA group for both the SM (*r* = 0.69, *p* < 0.01) and SMI (*r* = 0.62, *p* < 0.01) tasks. No significant effects were observed in individuals with WS (SM task: *r* = 0.26, *p* = 0.19; SMI task: *r* = 0.15, *p* = 0.46) (Figure 8). With respect to egocentric-bias errors, we observed a significant negative correlation with the SMI (*r* = −0.44, *p* < 0.01), but not the SM task in the VMA group (*r* = −0.31, *r* = 0.13). We did not observe any significant correlations in the WS group (SM task: *r* = −0.26, *p* = 0.19; SMI task: *r* = −0.27, *p* = 0.19) (Figure 9).

We found similar correct responses and egocentric-bias error patterns between the MR and VPT2 tasks in Experiment 1, and between the SM and SMI tasks in Experiment 2. Thus, it is possible that MR and SM tasks engage similar mental processes. However, the results of the developmental trajectory analysis of the WS group indicated that, while MR performance significantly improved, SM performance did not. Therefore, we directly compared MR and SM task performance and found that the SM task performance was significantly worse than MR task performance in the WS group (*p* < 0.001), but not in the VMA (*p* = 0.06) and CA (*p* = 0.89) groups. We also directly compared VPT2 and SMI task performance and found that performance on the SMI task was significantly better than that on the VPT2 task in the VMA group (*p* < 0.01). This was not the case for the WS (*p* = 0.20) and the CA groups (*p* = 0.17). This data was affected by the fact that performance in the CA group for both tasks reached a ceiling level while performance in the WS group for both tasks was at chance level.

## Discussion

To the best of our knowledge, the current study is the first to investigate both MR and VPT2 task performance in individuals with WS, while considering developmental changes and the potential mechanisms that lead individuals with WS to exhibit impaired performance on the VPT2 task.

In Experiment 1, we found that people with WS performed poorly on MR and VPT2 tasks compared with normal controls. In terms of developmental trajectory, we found that in people with WS, MR task performance improved significantly with development, while VPT2 task performance did not. In Experiment 2, we manipulated the physical location of participants to investigate the source of difficulties that people with WS experience when completing VPT2 tasks. We introduced two experimental conditions: a self-motion task and a self-motion-imagery task. We found that both SM and SMI task performance was lower in the WS group than in control individuals. Moreover, task performance in the WS group did not improve with development, in contrast with the results of the control group.

Our findings can be summarized in three main points. First, the mental processes involved in the MR and VPT2 tasks were distinct, while the requirements of the VPT2 task were related to performance on the ToM tasks, as previously reported. Second, while processes related to MR tasks tend to develop slowly, processes related to VPT2 tasks seem to be impaired in individuals with WS (Experiment 1). Third, the poor VPT2 task performance previously observed in people with WS appears to be due to difficulty transitioning between the participant's perspective and a third-person perspective, and may also involve defective mental simulation of one's own body motion (Experiment 2).

Concordant with previous studies that investigated MR task performance in individuals with WS (Farran et al., 2001; Stinton et al., 2008), MR task performance was poor in people with WS compared with control individuals. As in a previous study that used a geometric figure with various orientations (Stinton et al., 2008), we found that performance in the VMA group was better than that in the WS group. However, contrary to the findings of Stinton et al. (2008), our results indicated that MR task performance in individuals with WS was significantly above chance. This discrepancy may be due to the fact that Stinton et al. (2008) used geometric shapes, which may have been less familiar to participants, while we used more familiar objects, such as toy animals, dolls, and cars. This discrepancy in familiarity may be related to differences in the amount of attention that the participants gave to the objects. As Hamilton et al. (2009) pointed out, the current task was relatively easy; that is, it consisted simply of pointing to one of four pictures. This minimized the need for verbal ability (Huttenlocher and Presson, 1973). Thus, the current task might have required a cognitive load that was lower than that of the task used by Stinton et al. (2008). This may have resulted in more attention being directed at the target objects, leading to better performance compared with the previous findings.

The correct responses in the VPT2 task were not significantly better than chance for the WS group, but were significantly better than chance in the VMA group. We adopted an experimental paradigm used by Hamilton et al. (2009), and so it is not surprising that performance in the VPT2 task in the VMA group was similar to their findings from children aged 6–10 years, whose performance was significantly above chance (Hamilton et al., 2009). Studies that have used a more complex VPT2 task or an appearance-reality task have reported that children do not reliably perform well until approximately 5–6 years of age (Flavell et al., 1986). Thus, VPT2 task performance seems to be task-dependent.

As Hamilton et al. (2009) noted, “the relationship between VPT2 and mentalizing supports the idea that the VPT2 should be considered a mentalizing task.” Further analysis in our study revealed that VPT2 task performance reflected ToM task performance in the VMA group, but not in the WS group. Additionally, VPT2 task performance in VMA children who passed the ToM tasks was significantly better than that in VMA children who failed the tasks. However, this difference was not observed for the MR task. Contrary to the results from the VMA group, we found a significant difference on the MR task, but not on the VPT2 task, in the WS group. This may be due to the overall low performance of WS participants on the VPT2 task. As a result, no significant effects were observed, in contrast with the findings from the MR task. Moreover, as we did not find a clear interaction between ToM task performance and SM/SMI task performance in Experiment 2, it appears that neither task is sensitive to ToM task performance.

Therefore, our findings indicate that mentalizing ability might be impaired among some individuals with WS. This interpretation supports the view that socio-cognitive impairments are a component of WS (Tager-Flusberg and Sullivan, 2000). It should be noted that we found only two participants in the WS group who received a nearly perfect score (5 points) in the VPT2 task (Figure 3) and successfully completed both the location change and unexpected contents tasks. Concordant with this view, Porter et al. (2008) reported a specific deficit in social understanding within one of two WS subgroups using a non-verbal version of the ToM task. This deficit was observed even when the effects of mental or chronological age were controlled.

The developmental trajectory approach (Thomas et al., 2009) revealed differential developmental differences between the MR and VPT2 tasks in the VMA and WS groups. Whereas task success in the VMA group significantly improved with development in both tasks, in the WS group, development only improved MR task performance. Because both tasks were closely matched in terms of task difficulty (Hamilton et al., 2009), these findings suggest distinct mental processes. In the WS group, the processes related to the MR task appear to develop slowly while those related to the VPT2 task remain impaired regardless of development.

Recent neuroimaging reports suggest that differential brain regions are activated during MR and VPT2 tasks. For instance, the right inferior parietal sulcus is involved in a MR task (Harris et al., 2000; Podzebenko et al., 2002; Harris and Miniussi, 2003; Zacks et al., 2003a; Zacks, 2008) and the TPJ region plays an important role in completing a VPT2 task (Zacks et al., 2003b; Samson et al., 2004, 2005; Aichhorn et al., 2006; Santiesteban et al., 2012).

Considering the possibility of an abnormal dorsal stream in individuals with WS (Atkinson et al., 1997) in addition to the neuroimaging findings outlined above, it is plausible that the delayed development in MR task performance observed in our WS group may be associated with an atypical brain structure or atypical activation in dorsal brain regions. In line with this possibility, several studies have shown the existence of several atypical cortical structures in people with WS, such as reduced gray matter density in the superior parietal regions (Reiss et al., 2004; Eckert et al., 2005), including the intraparietal sulcus (Meyer-Lindenberg et al., 2004), bilateral reductions in sulcus depth in the intraparietal/occipitotemporal sulcus (Kippenhan et al., 2005), and prominent folding abnormalities in the dorsal parietal cortex (Van Essen et al., 2006). Atypical fractional anisotropy in the right superior longitudinal fasciculus, which is associated with deficits in visuospatial construction, has also been reported in WS individuals (Hoeft et al., 2007).

Although reduced activation has been reported in the inferior parietal cortices (Mobbs et al., 2007), there is little evidence of cortical abnormalities in the TPJ region in individuals with WS (Eckert et al., 2005). Therefore, the observed impaired VPT2 task performance of individuals with WS may be due to cortical abnormalities in other regions. A recent study showed that differential cortical regions, such as the right inferior frontal gyrus and the dorsomedial prefrontal cortex, are involved in spatial tasks concerning the location of the self (Mazzarella et al., 2013). Furthermore, as several studies suggest that spatial perspective taking is an embodied cognitive process (May, 2004; Zacks and Michelon, 2005; Keehner et al., 2006; Kessler and Thomson, 2010), it is possible that impaired VPT2 task performance is related to the defective mental simulation of one's own body motion. Supporting this view, the results of Experiment 2 clearly indicate that SMI task performance in people with WS is significantly worse than that of normal controls. This suggests that people with WS experience difficulty updating the mental representation of their own perspective as it relates to the imagery of their bodily motion. Furthermore, our direct comparison between performance in the MR and SM tasks revealed that individuals with WS also have difficulty updating the mental representation of their own perspective as it relates to their physical bodily motion. Concordant with our findings, Nardini et al. (2008) investigated developmental changes for both body- and environmental-based reference frames in individuals with WS. They found no developmental improvement in the participant-move (body-based frame of reference) condition, but did find developmental changes in the array-move (environment-based frame of reference) condition. Considering these findings, the difficulty in VPT2 task performance observed in people with WS might be due to impaired simulation of the motion of one's own body. As outlined above, neuroimaging literature has indicated that the left posterior parietal cortex (Creem et al., 2001) or supplementary motor areas (Wraga et al., 2005), insula, and hippocampus (Lambrey et al., 2012) are involved in imagined rotations of one's self. Further studies are required to address these points and explore the cognitive and neural mechanisms underlying the task of adopting the viewpoint of another person, as well as the simulation of movement of one's own body.

In addition to correct responses (Hamilton et al., 2009), we analyzed patterns of error responses and found that egocentric-bias errors were significant in both the VPT2 and SMI tasks compared with the MR and SM. We observed significant reductions in egocentric-bias errors with subsequent development in the VMA group, but not in the WS group. This finding seems to be concordant with initial observations in the literature, which suggest that children aged 4–6 years typically report their own perspective (Piaget and Inhelder, 1956). We speculate that the consistent egocentric-bias error found in the WS group might reflect executive dysfunction (Jawaid et al., 2012) because previous behavioral studies have reported a close relationship between executive function ability and the theory of mind (Frye et al., 1995; Hughes, 1998; Perner and Lang, 2000; Carlson and Moses, 2001; Perner et al., 2002; Kloo and Perner, 2003; Carlson et al., 2004; Sabbagh et al., 2006).

In conclusion, our findings can be summarized in three points. First, we found that VPT2 task performance was lower than MR task performance in individuals with WS, and both performance scores were lower than those of the control groups. Second, we observed delayed developmental improvement in MR task performance and consistently impaired VPT2 task performance, irrespective of development, in individuals with WS. Third, the findings of our second experiment indicate that difficulties faced by people with WS in terms of VPT2 task performance (Experiment 1) may be due to defective mental simulation of the motion of one's own body.

### Conflict of interest statement

The authors declare that the research was conducted in the absence of any commercial or financial relationships that could be construed as a potential conflict of interest.
